# The ERK MAPK Pathway Is Essential for Skeletal Development and Homeostasis

**DOI:** 10.3390/ijms20081803

**Published:** 2019-04-12

**Authors:** Jung-Min Kim, Yeon-Suk Yang, Kwang Hwan Park, Hwanhee Oh, Matthew B. Greenblatt, Jae-Hyuck Shim

**Affiliations:** 1 Department of Medicine, University of Massachusetts Medical School, Worcester, MA 01605, USA; JungMin.Kim@umassmed.edu (J.-M.K.); Yen.Yang@umassmed.edu (Y.-S.Y.); 2 Department of Orthopaedic Surgery, Yonsei University College of Medicine, Seoul 03722, South Korea; khpark@yuhs.ac; 3 Department of Pathology and Laboratory Medicine, Weill Cornell Medical College, New York, NY 10065, USA; hhoh80@gmail.com (H.O.); mag3003@med.cornell.edu (M.B.G.); 4 Li Weibo Institute for Rare Diseases Research, University of Massachusetts Medical School, Worcester, MA 01605, USA

**Keywords:** MAPK, MEK1, MEK2, ERK, osteoblast, osteopenia, cleidocranial dysplasia

## Abstract

Mitogen-activated protein kinases (MAPKs) are a family of protein kinases that function as key signal transducers of a wide spectrum of extracellular stimuli, including growth factors and pro-inflammatory cytokines. Dysregulation of the extracellular signal-regulated kinase (ERK) MAPK pathway is associated with human skeletal abnormalities including Noonan syndrome, neurofibromatosis type 1, and cardiofaciocutaneous syndrome. Here, we demonstrate that ERK activation in osteoprogenitors is required for bone formation during skeletal development and homeostasis. Deletion of *Mek1* and *Mek2*, kinases upstream of ERK MAPK, in osteoprogenitors (*Mek1^Osx^Mek2^−/−^*), resulted in severe osteopenia and cleidocranial dysplasia (CCD), similar to that seen in humans and mice with impaired RUNX2 function. Additionally, tamoxifen-induced deletion of *Mek1* and *Mek2* in osteoprogenitors in adult mice (*Mek1^Osx-ERT^Mek2^−/−^*) significantly reduced bone mass. Mechanistically, this corresponded to decreased activation of osteoblast master regulators, including RUNX2, ATF4, and β-catenin. Finally, we identified potential regulators of osteoblast differentiation in the ERK MAPK pathway using unbiased phospho-mass spectrometry. These observations demonstrate essential roles of ERK activation in osteogenesis and bone formation.

## 1. Introduction

Homeostasis in the skeletal system is maintained by coupling between bone-forming osteoblasts and bone-resorbing osteoclasts through a process of continual remodeling. Regulation of the anabolic component of this homeostasis occurs through the controlled hierarchical differentiation of skeletal stem cells, osteoprogenitors, and mature osteoblasts [1]. A number of critical signaling pathways, such as transforming growth factor-beta (TGFβ)/bone morphogenic protein (BMP) signaling, wingless-type MMTV integration site (WNT) signaling, and fibroblast growth factors (FGFs), regulate the lineage commitment of skeletal stem cells to osteoprogenitors and subsequent maturation of osteoprogenitors to osteoblasts [2].

As important mediators of cellular signaling, mitogen-activated protein kinases (MAPKs) are evolutionally-conserved serine/threonine kinases that regulate diverse cellular functions including cell proliferation, differentiation, and apoptosis [3]. MAPKs are part of a phospho-relay system in which the MAPK is phosphorylated and activated by an MAP2K that is, in turn, phosphorylated and activated by an MAP3K. MAPKs are also dynamically regulated by accessory proteins, such as scaffolds and phosphatases. This complexity allows MAPK pathways to serve diverse tissue-specific functions including skeletal development and homeostasis [3,4,5,6,7].

The canonical extracellular signal-regulated kinase (ERK) MAPKs have two isoforms, ERK1 (MAPK3) and ERK2 (MAPK1), both of which are highly expressed in osteoblast-lineage cells. ERK1 and ERK2 MAPKs are activated by MAP2K MEK1 (MAP2K1) and MEK2 (MAP2K2) through phosphorylation of activation loop residues Thr202/Tyr204 and Thr185/Tyr187, respectively [8,9,10]. Several studies have shown that the ERK MAPK pathway promotes osteoblast differentiation and bone formation in vitro and in vivo [11,12,13]. Accordingly, mice lacking *Erk1* and *Erk2* in the limb and head mesenchyme (*Erk1^−/−^Erk2^Prx1^*) display severe limb deformity and impaired skeletal mineralization [14]. Similarly, mice expressing a dominant-negative mutant of the Map2k *Mek1* in mature osteoblasts exhibit hypomineralization of clavicle and calvaria [5], similar to cleidocranial dysplasia (CCD) seen in human patients with loss-of-function mutations in *RUNX2* or mice with *Runx2* haploinsufficiency [15,16]. 

Our data demonstrate that ERK activation in osteoprogenitors is required for osteoblast differentiation and bone formation via control of osteoblast master regulators including RUNX2, ATF4, and β-catenin. We generated mice lacking *Mek1* and *Mek2* in osteoprogenitors (*Mek1^Osx^Mek2^−/−^*) by crossing mice with a conditional deletion of *Mek1* in osteoprogenitors (*Mek1^Osx^*) and mice with a germline deletion of *Mek2* (*Mek2^−/−^*), demonstrating that inactivation of ERK in osteoprogenitors results in severe bone loss in both axial and appendicular bones along with characteristic CCD phenotypes. Likewise, inducible, postnatal deletion of *Mek1* and *Mek2* in osteoprogenitors (*Mek1^Osx-ERT^Mek2^−/−^*) significantly reduced bone mass, suggesting that ERK activation in osteoprogenitors is important for bone formation during skeletal homeostasis. Finally, unbiased phospho-mass spectrometry using the post translational modification (PTM) scan direct technology was performed in *Mek1/2*-sufficient and -deficient osteoblasts to identify potential ERK MAPK substrates important for osteoblast differentiation. Future work will be needed to explore the roles of these putative ERK substrates in osteogenesis and bone formation.

## 2. Results

### 2.1. The ERK MAPK Pathway is Highly Activated in Osteoblasts In Vitro and In Vivo

**Figure 1:**
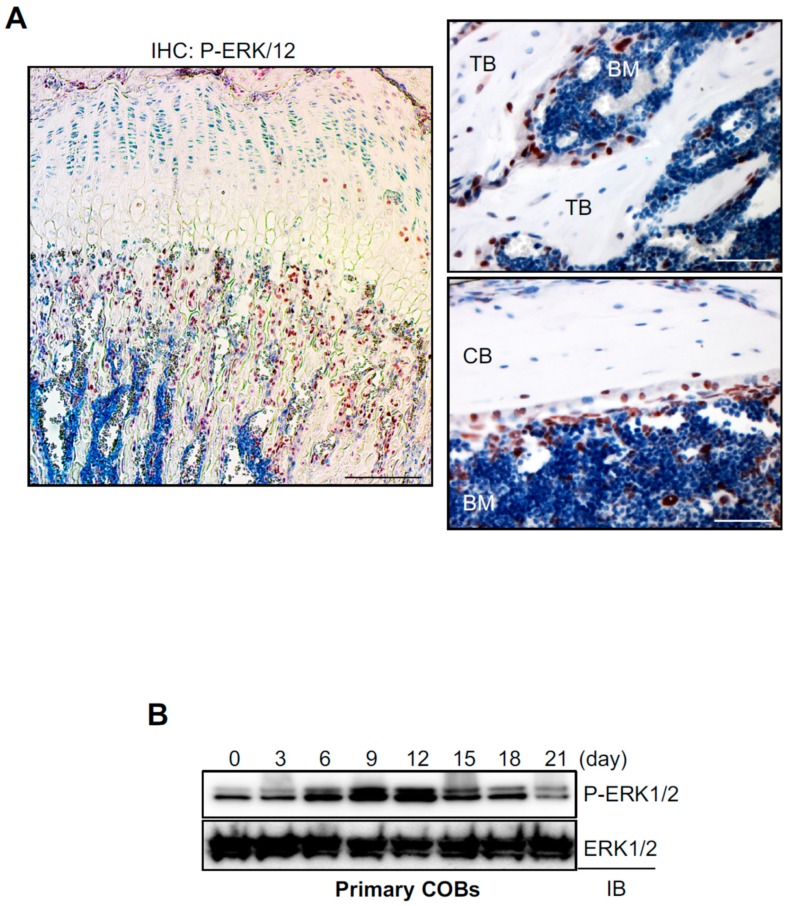
ERK activation in osteoblasts in vitro and in vivo. (**A**) Immunohistochemistry for phospho-ERK1/2 was performed in the femur of eight-week-old male mice. TB, trabecular bone; CB, cortical bone; BM; bone marrow. Scale bar, 500 µm (left) and 100 µm (right). (**B**) Primary calvarial osteoblasts (COBs) were isolated from mouse calvaria at Postnatal Day 5 and cultured under osteogenic conditions. Phosphorylation of ERK1/2 was determined by immunoblotting with anti-P-ERK1/2 antibody. ERK1/2 was used as a loading control.

To examine ERK activation in osteoblasts in vivo, immunohistochemistry was performed for phosphorylation of ERK1/2 in the femur of eight-week-old mice (Figure 1A). Phosphorylated ERKs were mainly detected in the trabecular bone area under the growth plate that has a high bone remodeling activity. Specifically, ERK1/2 were highly phosphorylated in osteoblasts on the bone surface, while osteocytes within the bone matrix showed modest phosphorylation of ERK1/2, suggesting dynamic ERK activation in osteoblast-lineage cells at different differentiation stages in vivo (Figure 1A). To test this hypothesis, we examined the kinetics of ERK phosphorylation during osteoblast differentiation in vitro (Figure 1B). Primary calvarial osteoblasts (COBs, osteoprogenitor) were isolated from mouse calvaria at Postnatal Day 5 (P5), cultured under osteogenic conditions for 21 days, and phosphorylation of ERK1/2 at different osteoblast differentiation stages was assessed by immunoblotting. Phosphorylation levels of ERK peaked at Day 12 of osteogenic culture and gradually decreased during later differentiation stages, demonstrating that ERK is highly activated in mature osteoblasts. Thus, these results suggest that ERK activation plays a role in osteoblast differentiation and function in vivo and in vitro.

### 2.2. Inactivation of ERK in Osteoprogenitors Causes Severe Osteopenia and Cleidocranial Dysplasia

**Figure 2:**
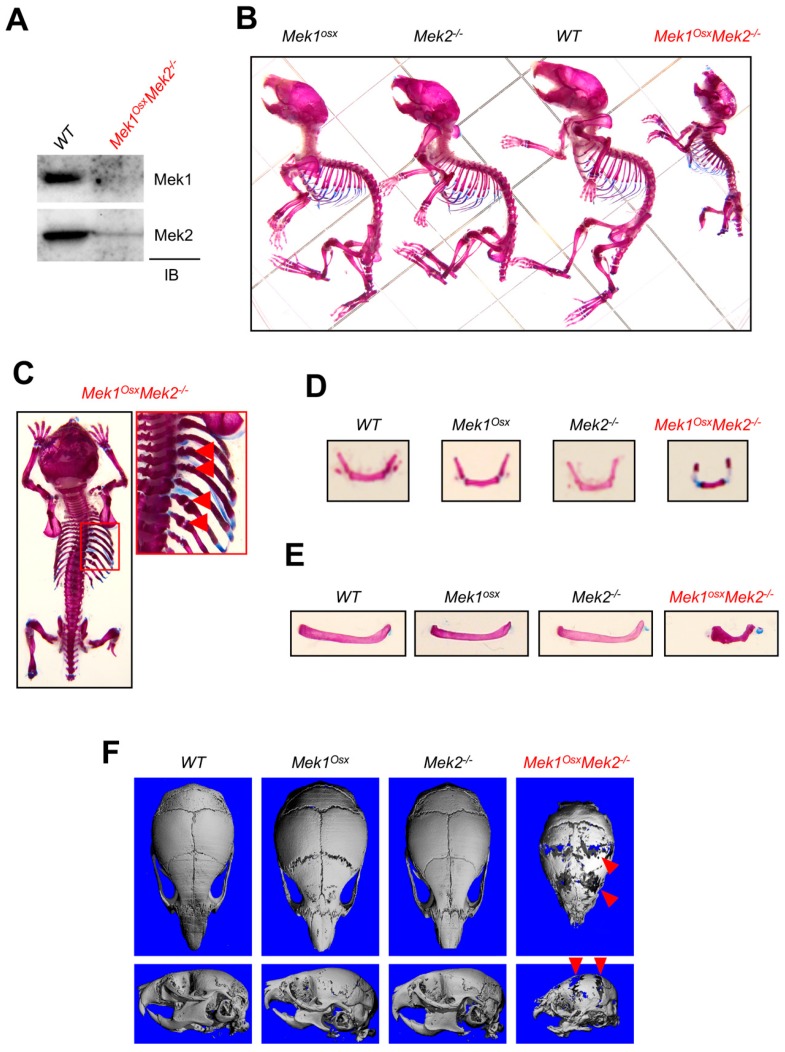
Inactivation of ERK in osteoprogenitors causes cleidocranial dysplasia (CCD) in mice. (**A**) Protein levels of Mek1 and Mek2 in the calvarium of WT and *Mek1^Osx^Mek2*^−/−^ neonates at Postnatal Day 5 (**B**–**E**) Alizarin red/Alcian blue staining of skeletal preparations of three-week-old WT, *Mek1^Osx^, Mek2*^−/−^, and *Mek1^Osx^Mek2*^−/−^ mice. The representative whole body images are displayed (**B,C**). The arrows indicate rib fractures in *Mek1^Osx^Mek2*^−/−^ mice (C, right). Hyoid bone (**D**) and clavicle (**E**) were also displayed. (**F**) MicroCT analysis shows representative 3D-reconstruction images of three-week-old WT, *Mek1^Osx^*, *Mek2*^−/−^, and *Mek1^Osx^ Mek2*^−/−^ calvaria. The arrows indicate hypomineralization areas.

**Figure 3:**
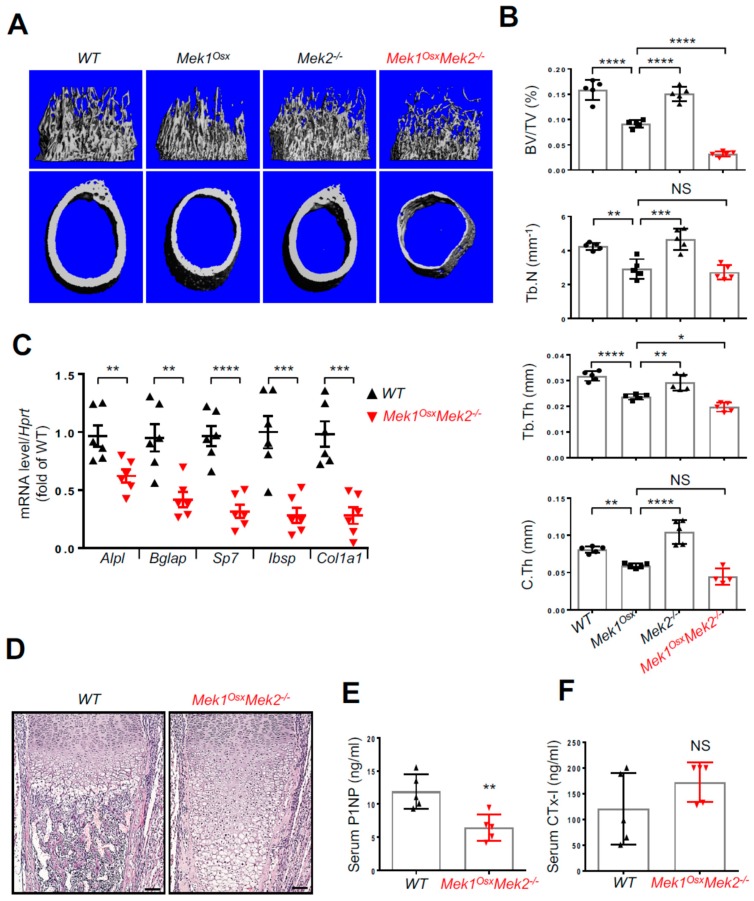
Inactivation of ERK in osteoprogenitors causes severe osteopenia in long bones. (**A,B**) MicroCT analysis of three-week-old WT, *Mek1^Osx^, Mek2*^−/−^, and *Mek1^Osx^Mek2*^−/−^ femurs. Representative 3D-reconstruction images of trabecular (upper) and cortical bone (lower) are displayed (**A**). Quantification of trabecular bone mass and midshaft cortical bone thickness are displayed (**B**). Trabecular bone volume/total volume (BV/TV), trabecular thickness (Tb.Th), trabecular number per cubic millimeter (Tb.N), and cortical thickness (C.Th). (*n* = 4~5). (**C**) Total RNAs were isolated from three-week-old WT and *Mek1^Osx^Mek2*^−/−^ tibias, and mRNA levels of osteoblast differentiation genes were measured by RT-PCR. (*n* = 6). (**D**) H&E-stained longitudinal sections of three-week-old WT and *Mek1^Osx^Mek2*^−/−^ femurs. Scale bar, 1 mm. (**E,F**) Serum levels of P1NP and CTx-I were measured by ELISA. (*n* = 5). Values represent the mean ± SD.; NS, not significant, * *p* < 0.05, ** *p* < 0.01, *** *p* < 0.001, and **** *p* < 0.0001 by the one-way ANOVA test (B) or an unpaired two-tailed Student’s *t*-test (C,E,F).

To investigate the role of the ERK MAPK pathway in osteoblasts in vivo, mice lacking *Mek1* and *Mek2*, mitogen-activated protein kinase kinases (MAP2Ks) upstream of ERK, in osteoprogenitors were generated by crossing mice harboring a floxed allele of *Mek1* (*Mek1^fl/fl^*) with Osx-Cre [17,18] in the *Mek2*-germline null background (*Mek2*^−/−^) [19,20]. Immunoblotting analysis validated deletion of Mek1 and Mek2 in the calvarium of P5 *Mek1^Osx^Mek2*^−/−^ neonates (Figure. 2A). While no obvious skeletal phenotypes were observed in mice with a deletion of *Mek1* (*Mek1^Osx^*) or *Mek2* (*Mek2*^−/−^) alone, *Mek1^Osx^Mek2*^−/−^ mice displayed severe growth retardation and skeletal defects and died at approximately four weeks old (Figure 2B). *Mek1^Osx^Mek2*^−/−^ mice were born at the expected Mendelian ratio. Skeletal preparations using Alizarin red/Alcian blue staining and microCT analysis of three-week-old *Mek1^Osx^Mek2*^−/−^ mice showed multiple rib fractures (Figure 2C), hypoplasia of the hyoid bone and clavicle (Figure 2D,E), and hypomineralization of the calvarium (Figure 2F). These phenotypes resemble those seen in human and murine cleidocranial dysplasia (CCD), characterized by open fontanelles, hypoplastic clavicles, and short stature [15,16]. Additionally, microCT analysis was performed in the femurs of WT, *Mek1^Osx^, Mek2*^−/−^*,* and *Mek1^Osx^Mek2*^−/−^ mice to assess bone mass (Figure 3A,B). While bone mass in *Mek2*^−/−^ femurs was comparable to that in wildtype femurs, *Mek1^Osx^* femurs displayed a modest decrease in trabecular bone mass and midshaft cortical thickness. When *Mek1* and *Mek2* were both deleted in osteoprogenitors, femoral trabecular bone mass, number, and thickness in addition to cortical thickness were all significantly reduced, demonstrating severe osteopenia (Figure 3A,B). Accordingly, expression of osteoblast differentiation genes, including alkaline phosphatase (*Alpl)*, osteocalcin (*Bglap)*, osterix (*Sp7)*, bone sialoprotein (*Ibsp)*, and type 1 collagen α1 (*Col1a1)*, was markedly decreased relative to WT femurs (Figure 3C). Histological analysis demonstrated that trabecular bone mass and cortical thickness were both reduced in *Mek1^Osx^Mek2*^−/−^ femurs, while bone marrow was filled with hypertrophic chondrocytes, suggesting delayed endochondral ossification and impaired osteoclast-mediated remodeling of the growth plate due to impaired expression of the Rank ligand (Figure 3D). Finally, serum levels of the bone formation marker, procollagen type I N-terminal propeptide (P1NP), and the bone resorption marker, C-terminal telopeptide of type I collagen (CTx-I), were measured in *Mek1^Osx^Mek2*^−/−^ mice, demonstrating a decrease in bone formation activity without any alteration in bone resorption activity (Figure 3E and F). Notably, these skeletal defects seen in *Mek1^Osx^Mek2*^−/−^ mice are more severe than those in mice with *Runx2* haploinsufficiency [15], suggesting that the ERK MAPK pathway has additional functions beyond RUNX2 regulation in osteoblasts. Taken together, ERK activation in osteoprogenitors is critical for skeletal development during the early postnatal stage.

### 2.3. Inducible Inactivation of the ERK Pathway in Osteoprogenitors Results in Osteopenia in Adult Mice

**Figure 4:**
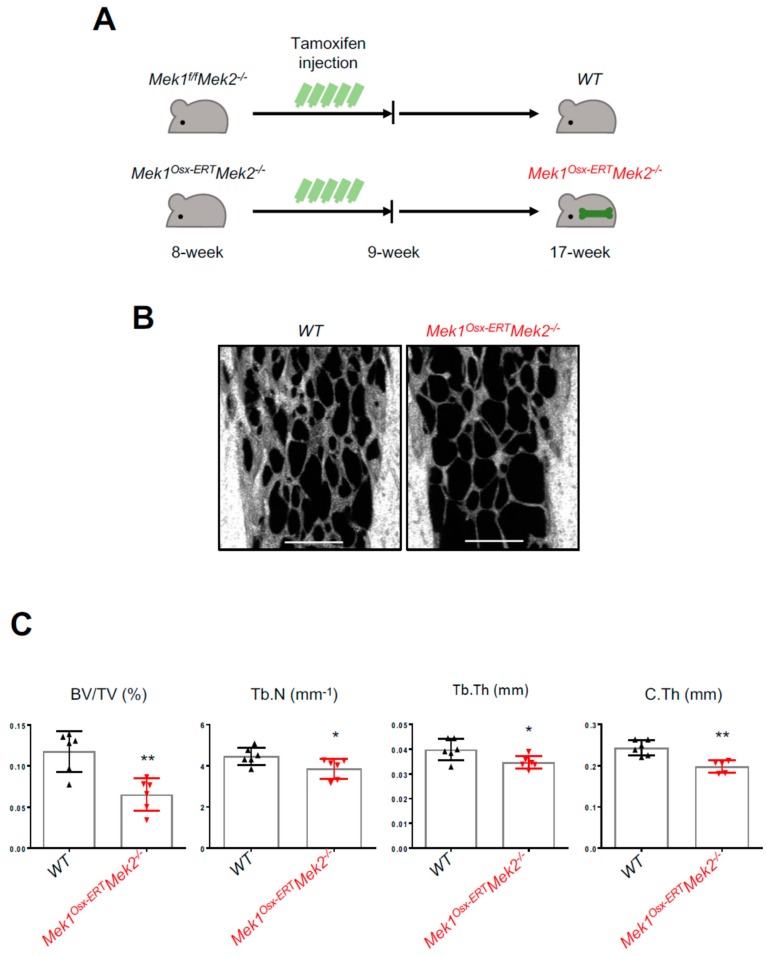
Inducible inactivation of ERK in osteoprogenitors decreases bone mass in adult mice. (**A**) A diagram of the strategy for tamoxifen-induced deletion of *Mek1* and *Mek2* in osteoprogenitors. (**B,C**) MicroCT analysis of 17-week-old male WT and *Mek1^Osx-ERT^Mek2*^−/−^ femurs. 3D-reconstruction images (B) and quantification (C) are displayed. Trabecular bone volume/total volume (BV/TV), trabecular thickness (Tb.Th), trabecular number per cubic millimeter (Tb.N), and cortical thickness (C.Th). (*n* = 5~6). Scale bar, 500 µm. Values represent mean ± SD.; * *p* < 0.05 and ** *p* < 0.01 by an unpaired two-tailed Student’s *t*-test (C).

To investigate the role of ERK activation in bone formation during later postnatal life, we generated an inducible, osteoblast-specific *Mek1/2*-knockout by crossing *Mek1^fl/fl^* mice with osterix-CreERT mice expressing a tamoxifen-inducible Cre recombinase in osteoprogenitors in the *Mek2*-germline null background (*Mek1^Osx-ERT^Mek2*^−/−^) [21]. Eight-week-old *Mek1^fl/fl^Mek2*^−/−^ and *Mek1^Osx-ERT^Mek2*^−/−^ mice were treated with tamoxifen for five consecutive days to induce Cre-mediated deletion of *Mek1* in osteoprogenitors, and nine weeks later, skeletal phenotypes were assessed by microCT (Figure 4A). Similar to *Mek1^Osx^Mek2*^−/−^ mice, trabecular bone mass, number, and thickness and midshaft cortical thickness were markedly reduced in *Mek1^Osx-ERT^Mek2*^−/−^ mice relative to those in *Mek1^fl/fl^Mek2^-/-^* mice (Figure 4B,C), demonstrating that ERK activation in osteoprogenitors is also important for bone formation during skeletal homeostasis.

### 2.4. The ERK MAPK Pathway Is Important for Activation of Osteoblast Master Regulators

**Figure 5:**
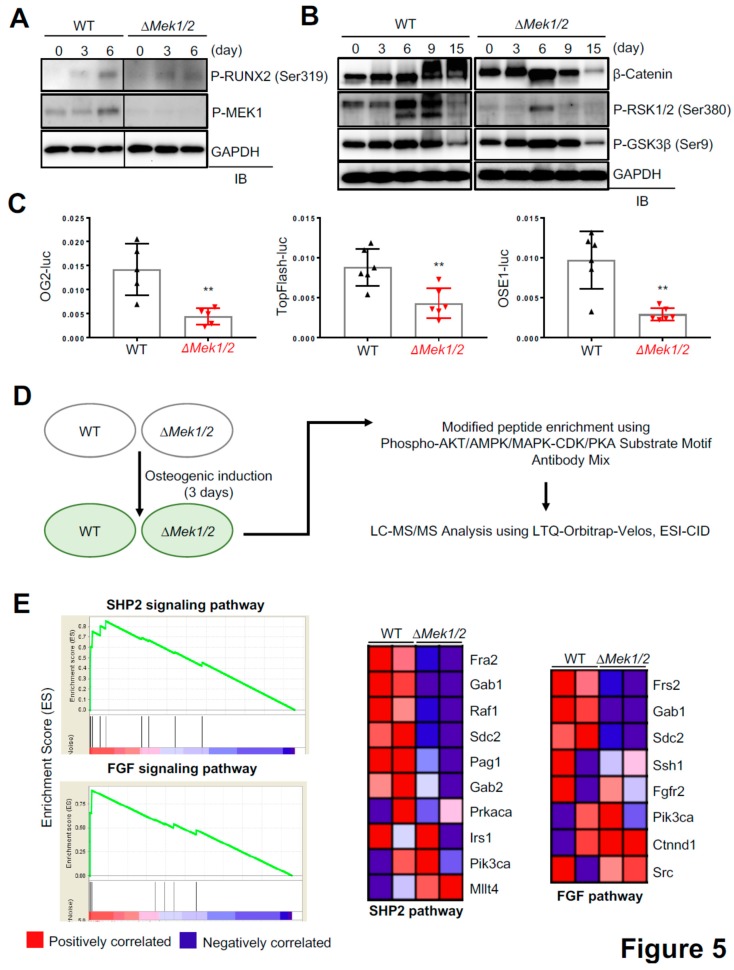
The MEK-ERK pathway is important for activation of osteoblast master regulators. (**A,B**) Primary WT and ∆*Mek1/2* COBs were cultured under osteogenic conditions and lysed at different time points. Phosphorylation levels of RUNX2 (Ser319) and MEK1 (A) and GSK3β (Ser9) and RSK1/2 (Ser380) (B) and protein levels of β-catenin (B) were assessed by immunoblotting with the indicated antibodies. (**C**) Primary WT and ∆*Mek1/2* COBs were transfected with OG2-luc (RUNX2), TopFlash-luc (Wnt/β-catenin), or OSE1-luc (ATF4) reporter genes along with *Renilla*. Twenty four hours after transfection, luciferase activity was measured and normalized to *Renilla.* (**D**) Experimental strategy to identify proteins differentially phosphorylated in WT and ∆*Mek1/2* COBs. (**E**) Enrichment plots (left) and gene signature sets (right) in WT and ∆*Mek1/2* COBs. Gene-set enrichment analysis (GSEA) analysis displays enrichment of genes involved in SHP2 and the FGF signaling pathway. Values represent mean ± SD.; ** *p* < 0.01 by an unpaired two-tailed Student’s *t*-test (C).

Our finding that inactivation of ERK in osteoprogenitors causes CCD phenotypes in mice implies that ERK plays a critical role in the regulation of RUNX2 in osteoblasts (Figure 2). Accordingly, a previous study has demonstrated that ERK1/2-mediated phosphorylation of RUNX2 at Ser319 is required for RUNX2 transcriptional activity and osteoblast differentiation [22]. In addition to RUNX2, two key regulators of osteoblast differentiation, ribosomal s6 kinase 1/2 (RSK1/2) that regulates ATF4 transcription activity [23,24] and GSK3β that regulates β-catenin stability [25,26,27], have been also reported as ERK substrates. To examine the ability of ERK to activate these regulators in osteoblasts, *Mek1-* and *Mek2*-deficient osteoblasts (∆*Mek1/2*) were generated by deleting *Mek1* in primary *Mek1^fl/fl^Mek2*^−/−^ COBs via lentivirus-mediated delivery of Cre recombinase. WT and ∆*Mek1/2* COBs were cultured under osteogenic conditions, and phosphorylation levels of RUNX2 (Ser319), RSK1/2 (Ser380), and GSK3β (Ser9) were assessed by immunoblotting (Figure 5A,B). In WT COBs, phosphorylation of MEK1, RUNX2, and RSK1/2 peaked at Day 6 of osteogenic culture, but were all markedly reduced in the absence of *Mek1/2* (∆*Mek1/2*) (Figure 5A). Intriguingly, ∆*Mek1/2* COBs showed a significant decrease in protein levels of β-catenin at Days 9 and 15 of osteogenic culture, while the kinetics of GSK3β phosphorylation in ∆*Mek1/2* COBs were comparable to those in wildtype COBs (Figure 5B), suggesting that ERK-mediated mechanisms independent of GSK3β phosphorylation are responsible for β-catenin stability in osteoblasts. These results are consistent with luciferase assays showing that the transcriptional activities of RUNX2 (OG2-luc), β-catenin (TopFlash-luc), and ATF4 (OSE1-luc) were all markedly decreased in ∆*Mek1/2* COBs (Figure 5C). Thus, the ERK MAPK pathway is critical for activation of RUNX2, β-catenin, and ATF4 in osteoblasts.

To identify novel regulators of osteoblast differentiation in the ERK MAPK pathway, unbiased phospho-mass spectrometry using the post translational modification (PTM) scan direct technology was performed in WT and ∆*Mek1/2* COBs (Figure 5D). Three days after osteogenic culture, cell lysates were immunoprecipitated using a mixture of antibodies specific to phosphorylated proteins regulated by MAPK, CDK, PKA, AKT, and AMPK, and the immunoprecipitates were subjected to mass spectrometry. Gene-set enrichment analysis (GSEA) using the intensity of identified proteins showed an enrichment of phosphorylated proteins in the pathways of SHP2 (encoded by *Ptpn11*), FGF (fibroblast growth factor), and SCF-KIT (stem cell factor-kit) in WT COBs, and that phosphorylation of the proteins in these pathways was markedly decreased in ∆*Mek1/2* COBs (Figure 5E and Supplementary Figure S1). The protein-tyrosine phosphatase SHP2 has been known to function upstream of MEK1/2 MAP2Ks and to be important for osteoblast maturation [28]. Additionally, FGF signaling activates the ERK MAPK pathway and functions as a key regulator of osteoblast differentiation and skeletal development [12,29]. Finally, the SCF-KIT pathway is found to protect osteoblasts from oxidative stress through activating c-Kit-AKT signaling [30]. However, further study is needed to understand the contribution of these pathways to the overall functions of the ERK pathway in osteoblasts. 

## 3. Discussion

In this study, we demonstrated that the ERK MAPK pathway is required for bone formation during skeletal development and homeostasis. Inactivation of ERK in osteoprogenitors (*Mek1^Osx^Mek2*^−/−^) at an early postnatal stage of skeletal development results in severe osteopenia and CCD phenotypes. Similarly, inducible, postnatal inactivation of ERK in osteoprogenitors (*Mek1^Osx-ERT^Mek2*^−/−^) significantly decreased bone mass. These results suggest that ERK activation plays a critical role in both skeletal development and homeostasis. Mechanistically, this corresponded to decreased activation of RUNX2, WNT/β-catenin, and RSK-ATF4 signaling pathways. Furthermore, unbiased phospho-mass spectrometry was performed to identify potential new regulators of osteoblast differentiation in the ERK MAPK pathway. 

The ERK MAPK pathway has been implicated as a positive regulator for osteoblast differentiation and bone formation [11,13,14]. A previous study has demonstrated that dominant-negative *Mek1* expression in osteoblasts results in CCD in mice, while constitutively-active *Mek1* expression in osteoblasts partially rescues CCD phenotypes caused by *Runx2* haploinsufficiency [5], suggesting that the MEK-ERK MAPK pathway regulates RUNX2 in osteoblasts. Consistent with this study, CCD phenotypes were also observed in mice lacking *Mek1* and *Mek2* in osteoprogenitors (*Mek1^Osx^Mek2*^−/−^) (Figure 2). Additionally, *Mek1^Osx^Mek2*^−/−^ mice showed delayed ossification of long bones along with increased number of hypertrophic chondrocytes (Figure 3D), a phenotype similar to that seen in *Erk1*^−/−^*Erk2^Osx^* mice [31]. 

ERK has several other known substrates besides RUNX2 that contribute to osteogenesis including, RSK2 [23]. RSK2 phosphorylates and activates the transcription factor ATF4, which is required for procollagen gene transcription in the later stages of osteoblast differentiation [24]. Additionally, ERK interacts with a wide range of parallel signal-transduction pathways in osteoblasts, such as the WNT/β-catenin pathway. ERK-mediated phosphorylation of GSK3β at Ser9 decreases its kinase activity, resulting in β-catenin accumulation through a reduction in ubiquitin-mediated proteasomal degradation [27]. Our data demonstrate that inactivation of ERK in osteoblasts leads to a significant decrease in the phosphorylation of RUNX2 and RSK2 and the corresponding transcriptional activity of RUNX2 and ATF4 (Figure 5A–C). Intriguingly, while GSK3β phosphorylation was not altered in the absence of *Mek1* and *Mek2*, ∆*Mek1/2* COBs displayed a substantial decrease in protein levels and transcription activity of β-catenin, implicating additional ERK-mediated mechanisms that regulate β-catenin stability in osteoblasts. 

Our unbiased phospho-mass spectrometry revealed a high enrichment of phosphorylated proteins in the pathways of SHP2, FGF, and SCF-KIT in WT COBs, and that phosphorylation of the proteins in these pathways was markedly decreased in ∆*Mek1/2* COBs. Among these proteins, phosphorylation of a member of the FOS family, FRA2 (*FOSL2*) [32], was substantially reduced in the absence of *Mek1* and *Mek2*. FRA2 is important for skeletal mineralization, as overexpression of *Fra2* (*Fosl2*) in transgenic mice increased bone formation, though understanding the mechanism regulating FRA2 in osteoblasts requires further study [33]. Our data identified potential regulators of osteogenic differentiation and bone formation and will thereby improve understanding of ERK-mediated molecular mechanisms in osteogenesis and bone-related disease.

## 4. Materials and Methods

### 4.1. Antibodies and Cell Culture

Antibodies specific to phospho-ERK1/2 (Cell Signaling, 4376, Danvers, MA, USA), ERK2 (Cell Signaling, 4195), phospho-MEK1 (Cell Signaling, 9127), β-catenin (Cell Signaling, 9587), phospho-RSK1/2 (Santa Cruz, sc-12898, Santa Cruz, CA, USA), phospho-GSK3β (Cell Signaling, 9323), and GAPDH (Santa Cruz, sc-25778) were used according to the manufacturer’s instructions. Phospho-RUNX2 (Ser319) antibody was generated as previously described [34].

To generate ∆*Mek1/2* osteoblasts, primary calvarial osteoblasts (COBs) were isolated from calvaria of 5-day-old *MEK1^fl/fl^MEK2*^−/−^ neonates using collagenase type II (50 mg/mL, Worthington, LS004176)/dispase II (100 mg/mL, Roche, 10165859001, Mannheim, Germany), transduced with lentiviruses expressing cre-recombinase, and selected with puromycin. COBs were maintained in α-MEM medium (Gibco, Grand Island, NY, USA) containing 10% FBS (Corning, Corning, NY, USA), 2 mM L-glutamine (Corning), 1% penicillin/ streptomycin (Corning), and 1% nonessential amino acids (Corning) and differentiated with ascorbic acid (200 µM, Sigma, A8960, Saint Louis, MO, USA) and β-glycerophosphate (10 mM, Sigma, G9422).

### 4.2. Mice

*Mek1^fl/fl^* mice and *Mek2*^−/−^ mice were generated as previously reported, respectively [19,35], and maintained in a 129/SvEv background. To generate osteoprogenitor-specific double knockout (*Mek1^Osx^Mek2*^−/−^), mice were crossed with Osx-cre [17,18], and littermate control was used for all skeletal analyses. To generate mice harboring inducible deletion of *Mek1* in osteoprogenitors, *Mek1^fl/fl^Mek2*^−/−^ mice were crossed with Osterix-CreERT [21]. For postnatal activation of CreERT, 75 mg/kg of tamoxifen (Sigma, T5648) in corn oil (Sigma) were intraperitoneally injected into 8-week-old mice once a day for five consecutive days. All animals including Mek1-floxed mice and Mek2 knockout mice were used in accordance with the NIH Guide for the Care and Use of Laboratory Animals and were handled according to the animal protocol approved by the University of Massachusetts Medical School on animal care (IACUC).

### 4.3. MicroCT and Skeletal Preparation

MicroCT was used for qualitative and quantitative assessment of trabecular and cortical bone microarchitecture and performed by an investigator blinded to the genotypes of the animals under analysis. Femurs excised from the indicated mice were scanned using a microCT 35 (Scanco Medical, Brüttisellen, Switzerland) with a spatial resolution of 7 μm. Briefly, trabecular bone mass of the distal femur was measured in an upper 2.1-mm region beginning 280 μm proximal to the growth plate, and cortical bone thickness was measured in a midshaft region of 0.6 mm in length. MicroCT analysis of 3-week-old skulls was performed at isotropic voxel sizes of 12 μm. 3D reconstruction images were generated from contoured 2D images using microCT software (Brüttisellen, Switzerland). Alternatively, the Inveon multimodality 3D visualization program was used to generate fused 3D views of multiple static or dynamic volumes of microCT modalities (Siemens Medical Solutions USA, Inc., Norwood, MA, USA). All images presented are representative of the respective genotypes (*n* > 4).

For skeletal preparation, mice were skinned, eviscerated, and fixed in 95% EtOH for a day, and the skeletons were transferred in acetone for 2 days. Then, skeletons were stained with 0.1% of Alizarin red s and 0.3% of Alcian blue (Sigma, A3157) solution for 3 days as previously described [36]. After staining, samples were washed with 95% EtOH, and soft tissue was cleared by a 1.5% KOH solution. Soft tissue was subsequently further cleared in 1% KOH. All images presented are representative of the respective genotypes (*n* > 5).

### 4.4. Histology and Immunohistochemistry

For histological analysis, 3- or 8-week-old femurs were fixed in 10% neutral formalin at 4 °C for 2 days and then decalcified in 15% tetrasodium EDTA (pH 8.0) at 4 °C for 2 weeks. Tissues were dehydrated in different concentrations of EtOH, incubated in xylene, and embedded in paraffin. Paraffin sections were performed at a 7-µm thickness along the coronal plate and stained with hematoxylin and eosin (H&E).

For immunohistochemistry, longitudinal sections of 8-week-old femurs were stained with anti-phospho-ERK1/2 antibody (Cell Signaling, 4376). Briefly, after deparaffinization and rehydration, paraffin tissue sections were blocked with 3% goat serum, 1% BSA, 0.1% Triton X-100 in PBS for 1 h at room temperature, incubated with primary antibody specific to phospho-ERK1/2 overnight at 4 °C, followed by TSA-biotin (Perkin Elmer, Waltham, MA, USA) and streptavidin-HRP, and then visualized with 2,2′-diaminobenzidine tetrahydrochloride as per the manufacturer’s instructions. 

### 4.5. RT-PCR and Immunoblotting

Total RNAs were purified using QIAzol (Qiagen, Germantown, MA, USA), and cDNA was synthesized using the High-Capacity cDNA Reverse Transcription Kit (Applied Biosystems, Beverly, MA, USA). Quantitative RT-PCR was performed using SYBR^®^ Green PCR Master Mix (Bio-Rad, Hercules, CA, USA) with the Bio-Rad CFX Connect Real-Time PCR detection system. The primers used for PCR are described in the Supplementary Table S1.

For immunoblotting, cells were lysed in TNT lysis buffer (50 mM Tris, 50 mM NaCl, 1% Triton X-100, 1 mM EDTA, 10 mM NaF, proteinase inhibitor cocktails (Sigma, P8340)), subjected to SDS-PAGE, and transferred to Immobilon-P membranes (Millipore, Burlington, MA, USA). After incubation with a blocking buffer (5% skim milk in TTBS buffer (100 mM Tris-HCl, pH 7.5, 150 mM NaCl, 0.1% Tween-20), the membranes were treated with the indicated antibodies, incubated with HRP-conjugated secondary antibodies, and developed with ECL (Thermo Scientific, Waltham, MA, USA). Anti-GAPDH antibody was used as a loading control.

### 4.6. Luciferase Reporter Assay

WT and ∆*Mek1/2* COBs were transfected with the RUNX2-responsive reporter gene (OG2-luc), β-Catenin-responsive reporter gene (TopFlash-luc), or ATF4-responsive reporter gene (OSE1-luc) with the *Renilla* luciferase vector (Promega, Madison, WI, USA) using the Effectene transfection reagent (Qiagen). Twenty four hours after transfection, the dual luciferase assay was performed according to the manufacturer’s protocol (Promega), and luciferase activity was normalized to *Renilla*.

### 4.7. Phospho-Mass Spectrometry-Based Antibody Enrichment 

Post translational modification (PTM) scan direct technology from Cell Signaling Technology (CST, Danvers, MA, USA) was performed in WT and ∆*Mek1/2* COBs to identify phosphorylated proteins regulated by ERK. Briefly, two sets of WT and ∆*Mek1/2* COBs were cultured under osteogenic conditions for 3 days. Cell extracts were incubated with an antibody mixture (Phospho-Akt/AMPK/MAPK/CDK/PKA Substrate Motif Antibodies Mix, Cell Signaling, 9614/10001/759/2325/9624, respectively) and antibodies were immobilized to protein A (or G) agarose. After immunoprecipitation, eluted proteins were analyzed by LC-MS/MS using LTQ-Orbitrap-Velos, ESI-CID. PTM scan results in WT and ∆Mek1/2 COBs are shown in Supplementary Table S2. 

### 4.8. Gene Set Enrichment Analysis 

Proteins with more than two unique peptides derived from the parent ion intensity were defined as qualified proteins for GSEA analysis. Gene sets from the Broad Institute Molecular Signatures Database were used, and multiple lists of enriched gene sets were generated using the GSEA algorithm as previously described [37]. Enrichment for up-/down-regulated proteins in ∆*Mek1/2* COBs was assessed against a rank list of all the available expression values from ∆*Mek1/2* COBs to expression from WT COBs. The permutation type was set to phenotype, and other settings were set as default. A nominal *p*-value <0.05 and FDR (false discovery rate) *q*-value <0.25 were considered as a significantly enriched pathway.

### 4.9. Statistical Analysis

All data are shown as the mean ± standard deviation (SD). We first performed the Shapiro–Wilk normality test for checking normal distributions of the groups. If normality tests passed, two-tailed, unpaired Student’s *t*-tests, and if normality tests failed, Mann–Whitney tests were used for the comparisons between two groups. To compare four groups, one-way ANOVA was used if normality tests passed, and then Tukey’s multiple comparison test was used for all pairs of groups. Statistical analysis was performed using the GraphPad PRISM software (v7.0a, La Jolla, CA, USA). *p* < 0.05 was considered statistically significant. * *p* < 0.05, ** *p* < 0.01, *** *p* < 0.001, **** *p* < 0.0001.

## 5. Conclusion

In this study, we demonstrated ERK MAPK pathway is required for skeletal development and bone homeostasis through analysis of genetic deletion of ERK upstream, *Mek1* and *Mek2* in osteoprogenitors in developmental stage (*Mek1^Osx^Mek2*^−/−^) and postnatal stage (*Mek1^Osx-ERT^Mek2*^−/−^). This study strongly supports previous studies on ERK MAPK function in osteoblast differentiation and bone formation, phenotypically and mechanistically. Moreover, unbiased phospho-mass spectrometry provides putative downstream of ERK MAPK pathway in osteoblast differentiation and further study will improve knowledge regarding the role of ERK MAPK in bone biology. 

## Supplementary Materials

Supplementary materials can be found at https://www.mdpi.com/1422-0067/20/8/1803/s1.

## References

[1] Long F. (2011). Building strong bones: Molecular regulation of the osteoblast lineage. Nat. Rev. Mol. Cell Biol..

[2] Chen Q., Shou P., Zheng C., Jiang M., Cao G., Yang Q., Cao J., Xie N., Velletri T., Zhang X. (2016). Fate decision of mesenchymal stem cells: Adipocytes or osteoblasts?. Cell Death Differ..

[3] Johnson G.L., Lapadat R. (2002). Mitogen-activated protein kinase pathways mediated by ERK, JNK, and p38 protein kinases. Science.

[4] Greenblatt M.B., Shim J.H., Glimcher L.H. (2013). Mitogen-activated protein kinase pathways in osteoblasts. Annu. Rev. Cell Dev. Biol..

[5] Ge C., Xiao G., Jiang D., Franceschi R.T. (2007). Critical role of the extracellular signal-regulated kinase-MAPK pathway in osteoblast differentiation and skeletal development. J. Cell Biol..

[6] Greenblatt M.B., Shim J.H., Zou W., Sitara D., Schweitzer M., Hu D., Lotinun S., Sano Y., Baron R., Park J.M. (2010). The p38 MAPK pathway is essential for skeletogenesis and bone homeostasis in mice. J. Clin. Investig..

[7] Morrison D.K., Davis R.J. (2003). Regulation of MAP kinase signaling modules by scaffold proteins in mammals. Annu. Rev. Cell Dev. Biol..

[8] Robinson M.J., Cheng M., Khokhlatchev A., Ebert D., Ahn N., Guan K.L., Stein B., Goldsmith E., Cobb M.H. (1996). Contributions of the mitogen-activated protein (MAP) kinase backbone and phosphorylation loop to MEK specificity. J. Biol. Chem..

[9] Murphy L.O., Blenis J. (2006). MAPK signal specificity: The right place at the right time. Trends Biochem. Sci..

[10] Shaul Y.D., Seger R. (2007). The MEK/ERK cascade: From signaling specificity to diverse functions. Biochim. Biophys. Acta.

[11] Xiao G., Jiang D., Thomas P., Benson M.D., Guan K., Karsenty G., Franceschi R.T. (2000). MAPK pathways activate and phosphorylate the osteoblast-specific transcription factor, Cbfa1. J. Biol. Chem..

[12] Xiao G., Jiang D., Gopalakrishnan R., Franceschi R.T. (2002). Fibroblast growth factor 2 induction of the osteocalcin gene requires MAPK activity and phosphorylation of the osteoblast transcription factor, Cbfa1/Runx2. J. Biol. Chem..

[13] Xiao G., Gopalakrishnan R., Jiang D., Reith E., Benson M.D., Franceschi R.T. (2002). Bone morphogenetic proteins, extracellular matrix, and mitogen-activated protein kinase signaling pathways are required for osteoblast-specific gene expression and differentiation in MC3T3-E1 cells. J. Bone Miner. Res..

[14] Matsushita T., Chan Y.Y., Kawanami A., Balmes G., Landreth G.E., Murakami S. (2009). Extracellular signal-regulated kinase 1 (ERK1) and ERK2 play essential roles in osteoblast differentiation and in supporting osteoclastogenesis. Mol. Cell. Biol..

[15] Otto F., Thornell A.P., Crompton T., Denzel A., Gilmour K.C., Rosewell I.R., Stamp G.W., Beddington R.S., Mundlos S., Olsen B.R. (1997). Cbfa1, a candidate gene for cleidocranial dysplasia syndrome, is essential for osteoblast differentiation and bone development. Cell.

[16] Mundlos S. (1999). Cleidocranial dysplasia: Clinical and molecular genetics. J. Med. Genet..

[17] Rodda S.J., McMahon A.P. (2006). Distinct roles for Hedgehog and canonical Wnt signaling in specification, differentiation and maintenance of osteoblast progenitors. Development.

[18] Ono N., Ono W., Nagasawa T., Kronenberg H.M. (2014). A subset of chondrogenic cells provides early mesenchymal progenitors in growing bones. Nat. Cell Biol..

[19] Bissonauth V., Roy S., Gravel M., Guillemette S., Charron J. (2006). Requirement for Map2k1 (Mek1) in extra-embryonic ectoderm during placentogenesis. Development.

[20] Scholl F.A., Dumesic P.A., Barragan D.I., Harada K., Bissonauth V., Charron J., Khavari P.A. (2007). Mek1/2 MAPK kinases are essential for Mammalian development, homeostasis, and Raf-induced hyperplasia. Dev. Cell.

[21] Maes C., Kobayashi T., Selig M.K., Torrekens S., Roth S.I., Mackem S., Carmeliet G., Kronenberg H.M. (2010). Osteoblast precursors, but not mature osteoblasts, move into developing and fractured bones along with invading blood vessels. Dev. Cell.

[22] Ge C., Xiao G., Jiang D., Yang Q., Hatch N.E., Roca H., Franceschi R.T. (2009). Identification and functional characterization of ERK/MAPK phosphorylation sites in the Runx2 transcription factor. J. Biol. Chem..

[23] Dalby K.N., Morrice N., Caudwell F.B., Avruch J., Cohen P. (1998). Identification of regulatory phosphorylation sites in mitogen-activated protein kinase (MAPK)-activated protein kinase-1a/p90rsk that are inducible by MAPK. J. Biol. Chem..

[24] Yang X., Matsuda K., Bialek P., Jacquot S., Masuoka H.C., Schinke T., Li L., Brancorsini S., Sassone-Corsi P., Townes T.M. (2004). ATF4 is a substrate of RSK2 and an essential regulator of osteoblast biology; implication for Coffin-Lowry Syndrome. Cell.

[25] Day T.F., Guo X., Garrett-Beal L., Yang Y. (2005). Wnt/beta-catenin signaling in mesenchymal progenitors controls osteoblast and chondrocyte differentiation during vertebrate skeletogenesis. Dev. Cell.

[26] Hill T.P., Taketo M.M., Birchmeier W., Hartmann C. (2006). Multiple roles of mesenchymal beta-catenin during murine limb patterning. Development.

[27] Ding Q., Xia W., Liu J.C., Yang J.Y., Lee D.F., Xia J., Bartholomeusz G., Li Y., Pan Y., Li Z. (2005). Erk associates with and primes GSK-3beta for its inactivation resulting in upregulation of beta-catenin. Mol. Cell.

[28] Lapinski P.E., Meyer M.F., Feng G.S., Kamiya N., King P.D. (2013). Deletion of SHP-2 in mesenchymal stem cells causes growth retardation, limb and chest deformity, and calvarial defects in mice. Dis. Model. Mech..

[29] Ornitz D.M., Marie P.J. (2015). Fibroblast growth factor signaling in skeletal development and disease. Genes Dev..

[30] Yang L., Wu Z., Yin G., Liu H., Guan X., Zhao X., Wang J., Zhu J. (2014). Stem cell factor (SCF) protects osteoblasts from oxidative stress through activating c-Kit-Akt signaling. Biochem. Biophys. Res. Commun..

[31] Chen Z., Yue S.X., Zhou G., Greenfield E.M., Murakami S. (2015). ERK1 and ERK2 regulate chondrocyte terminal differentiation during endochondral bone formation. J. Bone Miner. Res..

[32] Hess J., Angel P., Schorpp-Kistner M. (2004). AP-1 subunits: Quarrel and harmony among siblings. J. Cell Sci..

[33] Ruther U., Garber C., Komitowski D., Muller R., Wagner E.F. (1987). Deregulated c-fos expression interferes with normal bone development in transgenic mice. Nature.

[34] Ge C., Yang Q., Zhao G., Yu H., Kirkwood K.L., Franceschi R.T. (2012). Interactions between extracellular signal-regulated kinase 1/2 and p38 MAP kinase pathways in the control of RUNX2 phosphorylation and transcriptional activity. J. Bone Miner. Res..

[35] Belanger L.F., Roy S., Tremblay M., Brott B., Steff A.M., Mourad W., Hugo P., Erikson R., Charron J. (2003). Mek2 is dispensable for mouse growth and development. Mol. Cell. Biol..

[36] McLeod M.J. (1980). Differential staining of cartilage and bone in whole mouse fetuses by alcian blue and alizarin red S. Teratology.

[37] Zhong L., Zhou J., Chen X., Liu J., Liu Z., Chen Y., Bai Y. (2017). Quantitative proteomics reveals EVA1A-related proteins involved in neuronal differentiation. Proteomics.

