# Supplementary material for: The ERK MAPK Pathway Is Essential for Skeletal Development and Homeostasis
Source: Int J Mol Sci. 2019 Apr 12;20(8):1803. doi: 10.3390/ijms20081803 (PMC6514701; doi:10.3390/ijms20081803)
Supplement: Supplementary file 1 [file ijms-20-01803-s001.zip › ijms-481317 Supplementary Materials.pdf]

Supplementary Materials

The ERK MAPK pathway is essential for skeletal development and homeostasis

Jung-Min Kim[1], Yeon-Suk Yang[1], Kwang Hwan Park[3], Hwanhee Oh[4], Matthew B. Greenblatt[4], and Jae-Hyuck Shim[1,2]*

[1]Department of Medicine, [2]Li Weibo Institute for Rare Diseases Research, University of Massachusetts Medical School, Worcester, MA, USA

[3]Department of Orthopaedic Surgery, Yonsei University College of Medicine, Seoul, South Korea

[4]Department of Pathology and Laboratory Medicine, Weill Cornell Medical College, New York, NY, USA

Supplementary Table S1: Primer sequences used for RT-PCR.

Gene	Forward	Reverse
Mouse *Alpl*	CACAATATCAAGGATATCGACGTGA	ACATCAGTTCTGTTCTTCGGGTACA
Mouse *Ibsp*	CAGGGAGGCAGTGACTCTTC	AGTGTGGAAAGTGTGGCGTT
Mouse *Sp7*	ATGGCGTCCTCTCTGCTTGA	GAAGGGTGGGTAGTCATTTG
Mouse *Bglap2*	GCAGCACAGGTCCTAAATAG	GGGCAATAAGGTAGTGAACAG
Mouse *Col1a1*	ACTGTCCCAACCCCCAAAG	ACGTATTCTTCCGGGCAGAA
Mouse *Hprt*	CTGGTGAAAAGGACCTCTCGAAG	CCAGTTTCACTAATGACACAAACG

Supplementary Table S2: Post Translational Modification (PTM) scan results in WT and Δ*Mek1/2* COBs

Supplementary Figure S1. Enrichment of SCF-KIT signaling pathway in WT and Δ*Mek1/2* COBs.

Enrichment plots (left) and signature gene sets (right) in WT and *Mek1/2*-deficient (Δ*Mek1/2*) COBs culture. GSEA analysis displays enrichment of genes involved with SCF-KIT signaling pathway.

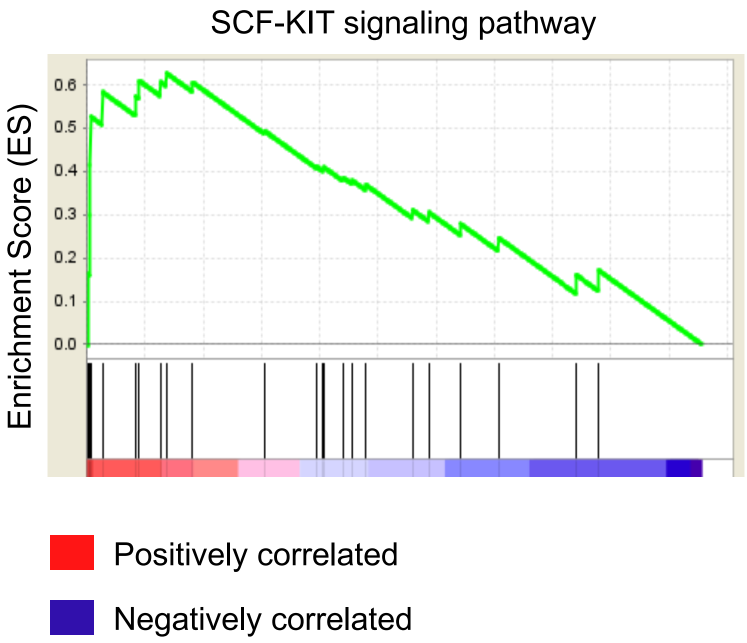

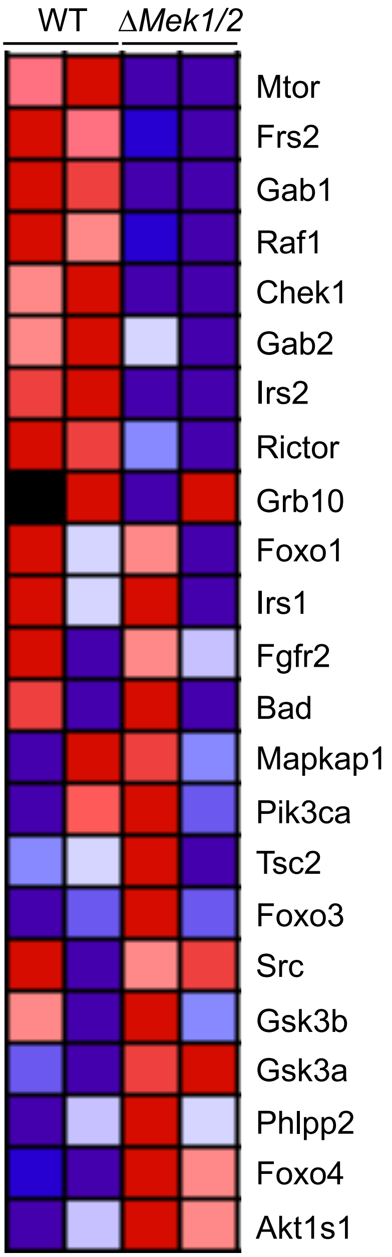

Supplementary Figure S1
